# Microbial therapeutics for acute colitis based on genetically modified *Lactococcus lactis* hypersecreting IL-1Ra in mice

**DOI:** 10.1038/s12276-020-00507-5

**Published:** 2020-09-28

**Authors:** Fu Namai, Suguru Shigemori, Tasuku Ogita, Takashi Sato, Takeshi Shimosato

**Affiliations:** Department of Biomolecular Innovation, Institute for Biomedical Sciences, Shinshu University, 8304 Minamiminowa, Kamiina, Nagano, 399-4598 Japan

**Keywords:** Immunology, Protein delivery

## Abstract

The increased incidence of inflammatory bowel disease (IBD) in Western and rapidly Westernizing developing countries poses a global pandemic threat. The development of affordable drugs for treating IBD worldwide is thus a priority. Genetically modified lactic acid bacteria (gmLAB) as microbial therapeutics are inexpensive protein producers suitable for use as carriers of protein to the intestinal mucosa. Here, we successfully constructed gmLAB hypersecreting interleukin 1 receptor antagonist (IL-1Ra). Oral administration of these gmLAB suppressed body weight reduction and exacerbation of the disease activity index score in mice with acute colitis and decreased the number of CD4^+^ IL-17A^+^ cells in the mesenteric lymph nodes. These data suggest that the gmLAB deliver IL-1Ra to the colon, where it inhibits IL-1 signaling. We thus developed a novel IBD therapeutic that blocks IL-1 signaling using a gmLAB protein delivery system. This system could be an inexpensive oral microbial therapeutic.

## Inflammatory bowel disease: Training bugs to deliver drugs

Genetically reprogrammed bacteria can facilitate the efficient delivery of a therapeutic protein for treating inflammatory bowel disease (IBD). Interleukin 1 receptor antagonist (IL-1Ra) inhibits the immune cells that attack the intestinal lining in IBD patients, but current administration strategies are associated with serious side effects. Takeshi Shimosato and colleagues at Shinshu University in Nagano, Japan, have engineered the microbe *Lactococcus lactis* to secrete high levels of IL-1Ra. The researchers dosed mice orally with these bacteria, which released IL-1Ra into the intestinal tissue. This treatment proved safe and effectively reduced inflammation and associated symptoms in a mouse model of ulcerative colitis. *L. lactis* has previously been tested as a probiotic in clinical trials, and may therefore offer an appealing alternative to subcutaneous administration of IBD drugs in human patients.

## Introduction

The increasing incidence of inflammatory bowel disease (IBD) is a problem worldwide, particularly in developing countries, thus raising the possibility of a serious global pandemic^1^. Considering the economic burden associated with IBD, the development of less expensive and easier-to-use therapeutics is an important issue. IL-1 signaling plays an important role in IBD immunity and inflammation. In IBD patients with Crohn’s disease and ulcerative colitis, mucosal mononuclear cells express IL-1β, and a positive correlation between severity and IL-1β expression has been demonstrated^2,3^. IL-1 signaling is regulated by endogenous IL-1 receptor antagonist (IL-1Ra), but an imbalance between IL-1 and IL-1Ra activity occurs in the gastrointestinal tract of IBD patients^4^. In a study using a murine IL-1Ra knockout model, deletion of IL-1Ra induced spontaneous IBD-like symptoms^4,5^. Therefore, inhibition of IL-1 signaling and correction of the agonist-antagonist imbalance in the inflamed portions of the intestinal tract are targets for the treatment of IBD. To date, several animal studies targeting IL-1 signaling using IL-1Ra or an anti-IL-1 antibody have been conducted and demonstrated an anti-inflammatory effect^6–8^. However, in clinical trials, serious side effects were reported in 5.1% of patients receiving subcutaneous administration of Anakinra, a recombinant human IL-1Ra^9^. Therefore, the delivery of IL-1Ra directly to the intestinal tract is desirable, as this approach might reduce the side effects and produce a beneficial anti-inflammatory effect^6^.

In this context, genetically modified lactic acid bacteria (gmLAB) delivery of IL-1Ra, the production of which does not require protein purification or sophisticated techniques for culture, could be an attractive strategy^10^. gmLAB are classified as microbial therapeutics and next-generation probiotics, and new opportunities for drug delivery using gmLAB are expected^11,12^. Indeed, compared with the oral administration of protein alone, an equivalent effect can be demonstrated with a smaller amount of protein when it is transported by gmLAB^13,14^. Here, we successfully constructed a microbial therapeutic based on genetically modified *Lactococcus* (*L*.) *lactis* hypersecreting recombinant mouse IL-1Ra (rmIL-1Ra) with the goal of facilitating a cost-effective treatment for IBD.

## Materials and methods

### Plasmid, bacteria, and growth conditions

A protein secretion vector plasmid for *L*. *lactis*, pNZ8148#2:SEC^15^, was used for recombinant gene expression. The sequence encoding mIL-1Ra optimized for *L. lactis* MG1363 was synthesized, and the sequence was cloned into pTAKN-2. *L. lactis* NZ9000 was purchased from MoBiTec (Goettingen, Germany) as the host and cultured at 30 °C in M17 broth with 0.5% glucose (GM17). gm–*L. lactis* NZ9000 was cultured in GM17 cm, which is GM17 medium with chloramphenicol (10 μg/mL).

### gmLAB for mIL-1Ra gene expression

Both ends of the mIL-1Ra gene encoded by pTAKN-2 were cleaved using the restriction enzymes *Hin*dIII and *Kpn*I (TaKaRa Bio, Inc., Tokyo, Japan). The resulting DNA fragment was ligated to pNZ8148#2:SEC digested with *Hin*dIII and *Kpn*I. The constructed plasmid (pNZ8148#2:SEC-IL1Ra) was subjected to sequence analysis (Eurofins Genomics) to examine consistency with the desired sequence. pNZ8148#2:SEC-IL1Ra was introduced into *L. lactis* NZ9000^15^ to generate the gmLAB (designated NZ-IL1Ra). Simultaneously, the original plasmid lacking the mIL-1Ra gene was also introduced into *L. lactis* NZ9000 to generate the vector control gmLAB (designated NZ–VC).

### Induction of recombinant gene expression and detection of rmIL-1Ra

Precultured gmLAB were inoculated into GM17 cm at a final concentration of 5% and incubated for 1–1.5 h (until the OD_600_ reached 0.4). Nisin was then added (final concentration 1.25 ng/mL) and incubated for 3 h. The culture was separated into cells and supernatant by centrifugation (8000 × *g*, 4 °C, and 1 min), and protein samples were prepared. Bacterial pellets were washed with Tris-buffered saline (TBS) and suspended in TBS + protease inhibitor cocktail (TBS + PIC). Glass beads (0.2 mmφ) were added to the solution, and the cells were crushed using a bead crusher. The resulting liquid of each sample was obtained by centrifugation (20,600 × *g*, 4 °C, and 15 min). A total of 300 µL of trichloroacetic acid was added to 1500 µL of supernatant sample and incubated for 1 h on ice to precipitate the protein. The precipitate was collected and washed twice with 400 µL of acetone. After centrifugation, the acetone was removed by incubation at 55 °C, and the precipitate was dissolved in 100 µL of 0.05 M NaOH.

Samples were separated by sodium dodecy sulfate polyacrylamide gel electrophoresis (SDS-PAGE), and the resolved proteins were stained with Coomassie Brilliant Blue (CBB) or transferred onto Amersham Hybond P PVDF membranes (GE Healthcare, Buckinghamshire, UK). The membranes were blocked for 1 h using skim milk and then incubated overnight with purified an anti-His Tag antibody (BioLegend, San Diego, CA, USA), followed by reaction for 1 h with an anti-mouse IgG (whole molecule) peroxidase-conjugated antibody (Sigma-Aldrich, St. Louis, MO, USA). Labeling was detected using ImageQuant LAS 500 (GE Healthcare).

rmIL-1Ra contained in the cell pellet lysate and supernatant was quantified by enzyme-linked immunosorbent assay (ELISA). The soluble fraction from the crushed cell pellet and supernatant collected from NZ-IL-1Ra incubated for 48 h was diluted using TBS + PIC and subjected to ELISA (Mouse IL-1Ra/IL-1F3 ELISA, R&D Systems, Minneapolis, MN, USA).

### Purification of rmIL-1Ra

NZ-IL1Ra was cultured (1 L), and rmIL-1Ra expression was induced as described above. The bacteria were pelleted by centrifugation (3000 × *g*, 4 °C, and 20 min). Supernatant-binding buffer (20 mM Na_3_PO_4_ 12 H_2_O, 500 mM NaCl, and 20 mM imidazole in the supernatant) was prepared and filtered (0.45 µm). After equilibrating a His-Trap HP 1-mL column with binding buffer, the filtrate was loaded, and the column was washed with binding buffer (20 column volumes [CVs]). The proteins adsorbed to the column were eluted with at least a 35-CV gradient of 0–500 mM imidazole at 1 mL/min using an AKTA Pure system (GE Healthcare). The obtained samples (supernatant, flow-through, wash, and eluate: E-1 to E-5) were subjected to SDS-PAGE and Western blotting. The resulting fraction (E-3) containing rmIL-1Ra was then dialyzed against phosphate-buffered saline (PBS) (10 mM disodium hydrogen phosphate, 2.7 mM potassium chloride, 137 mM sodium chloride, and 1.76 mM potassium dihydrogen phosphate [pH 7.4]). The concentration of rmIL-1Ra in the eluted fraction was determined by ELISA (Mouse IL-1Ra/IL-1F3, R&D Systems).

### Culture conditions for EL4.NOB-1 cells

Mouse ascites lymphoma lymphoblast EL4.NOB-1 cells that highly express IL-1R were purchased from Sigma-Aldrich and maintained in complete RPMI 1640 medium, which is RPMI 1640 (Sigma-Aldrich) containing fetal calf serum (10%, Sigma-Aldrich), streptomycin (100 mg/mL), penicillin (100 U/mL), HEPES (25 mM), nonessential amino acids, sodium pyruvate (1.0 mM), and 2-mercaptoethanol (0.0035%). EL4.NOB-1 cells were maintained at 37 °C with 5% CO_2_ and passaged every 3 days.

### Assay of rmIL-1Ra activity

EL4.NOB-1 cells (2 × 10^5^/170 µL) were plated in 96-well plates and incubated for 3 h. The preincubated EL4.NOB-1 cells were stimulated with IL-1β/IL-1F2 (200 pg/10 µL: R&D Systems) and various concentrations (37.25–250 ng/20 µL) of purified rmIL-1Ra, commercially available mIL-1Ra (R&D Systems), or commercially available human IL-1Ra (hIL-1Ra) (R&D Systems) for 24 h. After incubation, the supernatants of the EL4.NOB-1 cells were collected, and the concentration of IL-2 was assessed using the mouse IL-2 ELISA (R&D Systems).

### Intestinal delivery of rmIL-Ra by oral administration of NZ-IL1Ra

C57BL/6 mice (female, 7 weeks old) were purchased from Japan SLC (Shizuoka, Japan) and maintained for 2 weeks under controlled light and temperature conditions. The mice were provided a standard diet and autoclaved water *ad libitum*. The animal protocol was approved by the Animal Experiment Committee of Shinshu University (No. 240078).

Mice were separated into two groups (nonadministration group and NZ-IL1Ra administration group, *n* = 3–4). NZ-IL1Ra was cultured in a volume of 50 mL, and gene expression was induced as described above. NZ-IL1Ra was orally administered at 1.0 × 10^10^ CFU/200 µL every 30 min for 10 doses. Thirty minutes after the last oral administration, the mice were euthanized, and the serum and contents of the cecum and colon were collected. The cecal and colonic contents were suspended in PBS (200 mg/mL). The suspension was streaked onto a GM17 cm agar plate and incubated for 2 days. Eight of the resulting colonies were subjected to polymerase chain reaction (PCR) using the universal primers 27F (5′-AGAGTTTGATCCTGGCTCAG-3′) and 1492R (5′-GGCTACCTTGTTACGACTT-3′) to amplify the 16S rRNA region, and the resulting DNA fragments were sequenced and identified using BLAST. The supernatant of the cecum and colon content suspensions was obtained by centrifugation (20,600 × *g*, 4 °C, and 15 min). The mIL-1Ra concentration in the prepared sample was measured using the mouse IL-1Ra/IL-1F3 ELISA (R&D Systems).

### DSS-induced colitis mouse model protocol and oral administration

C57BL/6 mice (female, 7 weeks old) were purchased and maintained for 2 weeks as described above. After prehousing, mice were separated into two groups: NZ-VC (*n* = 18, DSS and NZ-VC treatment) and NZ-IL1Ra (*n* = 18, DSS and NZ-IL1Ra treatment). To induce acute colitis, mice were provided water supplemented with 3% DSS (MW = 36–50 kDa; MP Biomedicals, LLC, Solon, OH, USA) from day 0 to day 5. On day 5, the contents of the water bottle were switched to water alone until day 11. Body weight and disease activity index (DAI) scores were recorded daily^16^. For oral administration, gmLAB were cultured in a volume of 100 mL, and gene expression was induced as described above. gmLAB were suspended in PBS at 5.0 × 10^10^ CFU/mL, and mice were immediately administered 200 µL of the gmLAB suspension (1.0 × 10^10^ CFU) intragastrically for 12 consecutive days (days 0–11). On day 11, the mice were euthanized, the colon, mesenteric lymph nodes (MLNs), and cecal contents were immediately collected, and the colon length was measured.

### Histopathology

Distal colonic tissues were frozen in optimal cutting temperature compound and sliced using a Leica CM3050 S cryostat. The resulting sections were stained with HE. Tissue sections were observed using a BZ-X800 microscope (Keyence, Osaka, Japan). The total mucosal area was measured using the Hybrid Cell Count System in the BZ-X800 viewer (Keyence).

### Cytokine assay

Quantitative PCR (qPCR) was performed as described previously^17,18^. Primers for five genes (encoding IFN-γ, TNF-α, IL-1β, IL-6, and IL-17) were obtained from TaKaRa Bio, Inc. Alexa Fluor^®^ 488 anti-mouse CD4 (CAT# 100423; BioLegend) and PerCP/Cy5.5 anti-mouse IL-17A antibodies (CAT# 506919; BioLegend) were used. MLN cells were cultured in complete RPMI 1640 medium containing brefeldin A, ionomycin, and phorbol 12-myristate 13-acetate for 4 h. After fixation, cells were incubated with an Alexa Fluor^®^ 488 anti-mouse CD4 antibody for 15 min at 4 °C for surface staining. After permeabilization, cells were reacted with a PerCP/Cy5.5 anti-mouse IL-17A antibody for 60 min at 4 °C for intercellular staining. The percentage of CD4^+^ IL-17A^+^ cells was determined by flow cytometry. The acquired data were analyzed using FlowJo software (LLC, Ashland, OR, USA).

### Next-generation sequencing

Next-generation sequencing samples of day 11 cecal contents were prepared as previously described^19^. Sequencing quality control and trimming of the 341F and 806R forward and reverse primer sequences were conducted using DADA2^20^. Sequencing data were analyzed using the QIIME2 pipeline, including clustering, chimera checking, and α-diversity and β-diversity analyses^21^. The taxonomy assignment of each representative sequence was performed according to the tutorial on the QIIME2 website (https://qiime2.org) using the Greengenes database (Greengenes 13_8 99% operational taxonomic units [OTUs]).

### Statistical analysis

GraphPad Prism software (version 8, GraphPad, San Diego, CA, USA) was employed for statistical analysis, and significance was accepted at *p* < 0.05. For in vitro rmIL-1Ra bioactive assays, one-way ANOVA and Tukey–Kramer test were performed. For in vivo and related experiments, the ROUT method was used to identify outliers, and the data were then analyzed using unpaired *t* tests.

## Results

### *Lactococcus lactis* remarkably secretes rmIL-1Ra following nisin stimulation

**Fig. 1:**
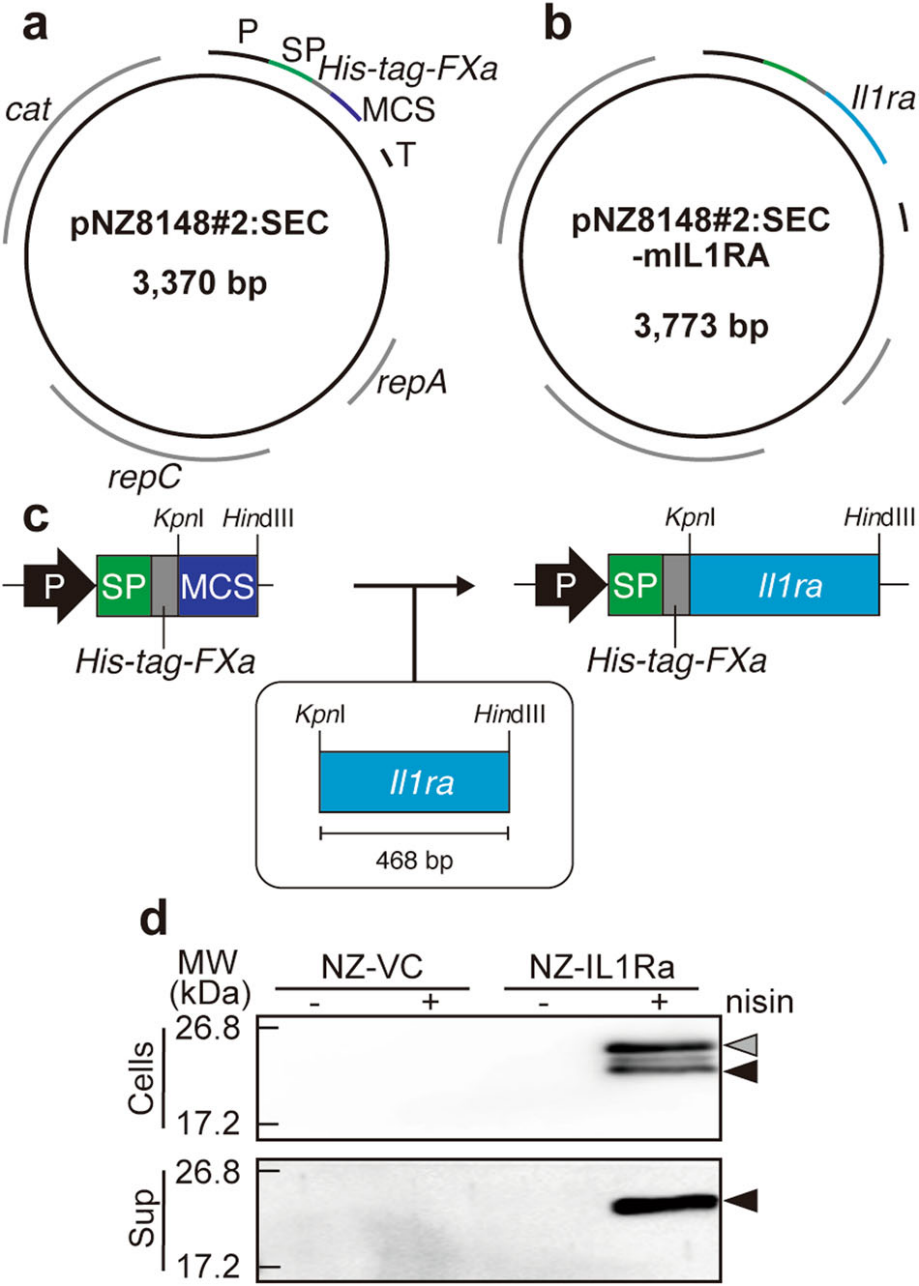
Construction of an IL-1Ra gene expression vector and analysis of rmIL-1Ra expression. **a** Vector map of the lactococcal secretion vector pNZ8148#2:SEC. P, nisin A-regulated promoter; SP sequence of the signal peptide from the USP45 protein, *His-tag* DNA sequence encoding the 6× histidine tag, *FXa* DNA sequence encoding the Factor Xa recognition site, MCS multiple cloning site, *T* terminator, *rep* replication gene, *cat* chloramphenicol acetyltransferase gene; **b** vector map of pNZ8148#2:SEC-IL1Ra. *mIL-1Ra*, the MG1363-optimized DNA sequence encoding mIL-1Ra. **c** Scheme of the procedure for constructing the IL-1Ra gene expression vector. **d** NZ–VC or NZ-IL1Ra was cultured in the presence (+) or absence (−) of nisin (1.25 ng/mL) for 3 h to induce recombinant protein expression. In the cell fraction, bands corresponding to the sizes of the SP and rmIL-1Ra complex (gray arrowhead: 24.5 kDa) and secretory rmIL-1Ra (black arrowhead: 21.8 kDa) were detected. A secreted rmIL-1Ra (black arrowhead: 21.8 kDa) band was also detected in the supernatant fraction.

pNZ8148#2:SEC-IL1Ra (Fig. 1b) was constructed by inserting sequences encoding mIL-1Ra into the multicloning site of pNZ8148#2:SEC (Fig. 1a, c). The plasmid was introduced into *L. lactis* NZ9000 to generate vector control gmLAB (NZ–VC) or gmLAB that secrete rmIL-1Ra (designated NZ-IL1Ra). Upon nisin stimulation, a band corresponding to pre-rmIL-1Ra was detected from the NZ-IL1Ra cell pellet by Western blotting (24.5 kDa, NZ-IL1Ra (+), Fig. 1d). In contrast, no bands from NZ-VC and NZ-IL1Ra without nisin were detected. In the supernatant of NZ-IL1Ra stimulated with nisin, a band corresponding to secreted rmIL-1Ra was detected (21.8 kDa, NZ-IL1Ra(+), Fig. 1d), but this band was not detected in the supernatant of NZ-VC and NZ-IL1Ra-nisin(−). These results clearly indicate that NZ-IL1Ra secretes rmIL-1Ra in a nisin stimulation-dependent manner. The yield of recombinant protein was as follows: cell pellet 100.75 ± 23.4 µg/mg and supernatant 2.00 ± 0.73 mg/L, immense quantities of rmIL-1Ra. We successfully obtained gmLAB that hypersecrete rmIL-1Ra in a nisin stimulation-dependent manner.

### Purification and bioactivity of rmIL-1Ra

**Fig. 2:**
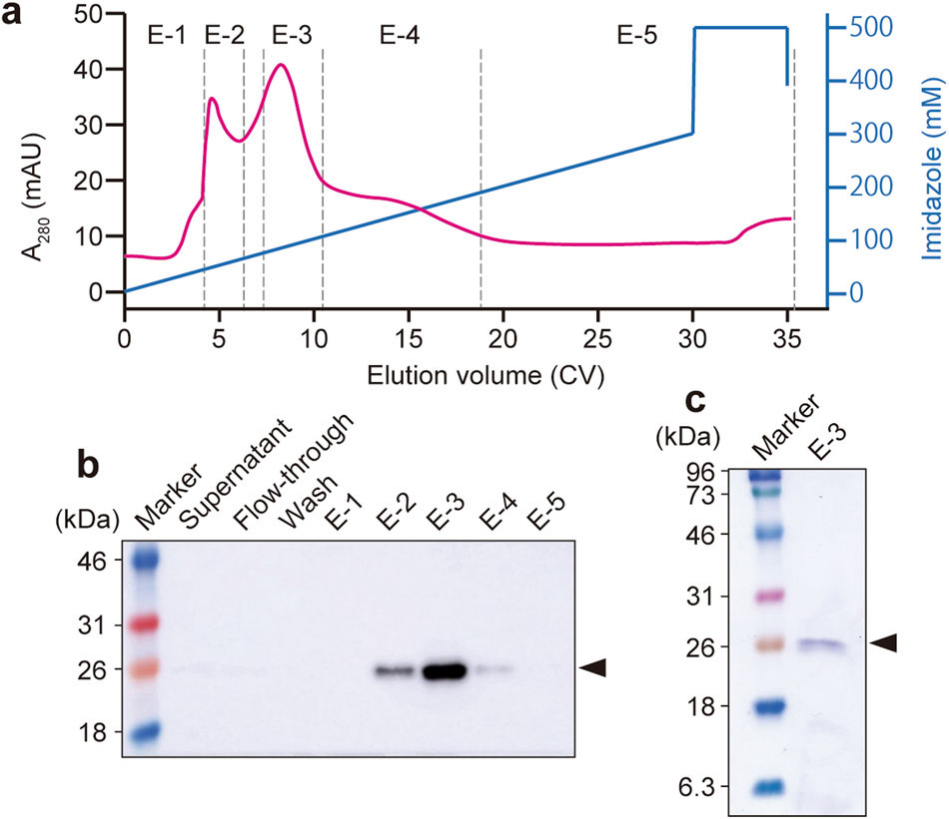
Purification of rmIL-1Ra. **a** Chromatogram of protein elution. Protein adsorbed on a His-Trap column was eluted by adding imidazole. The eluted protein was collected in five fractions (E-1 to E-5) based on A_280_. **b** The presence of rmIL-1Ra in each fraction was analyzed by Western blotting using an anti-His-tag antibody. **c** Results of analysis of E-3 by SDS-PAGE (CBB staining). Secretory rmIL-1Ra (black arrowhead: 21.8 kDa).

rmIL-1Ra was purified from the NZ-IL1Ra supernatant, and a chromatogram of the absorbance at 280 nm (A_280_) is shown in Fig. 2a. Based on the absorbance value, the purified solution was separated into five fractions (E-1 to E-5). The purity was assessed by SDS-PAGE followed by staining with CBB (Fig. 2c) or Western blotting (Fig. 2b). Bands corresponding to secreted rmIL-1Ra (21.8 kDa) were confirmed in E-2 to E-4, confirming that rmIL-1Ra was highly purified, particularly in fraction E-3. Therefore, in future experiments, E-3 was used as purified rmIL-1Ra. ELISA indicated that E-3 contained 50 µg of rmIL-1Ra.

**Fig. 3:**
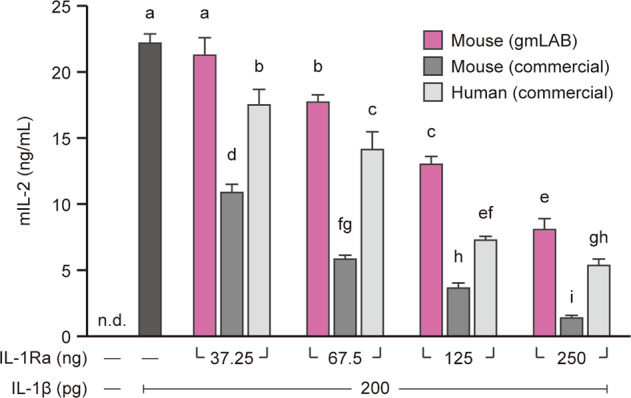
Assay of the bioactivity of rmIL-1Ra purified from NZ-IL1Ra cellular extract. EL4.NOB-1 cells (2 × 10^5^) were stimulated with IL-1β (200 pg/well) and various concentrations (37.25–250 ng/well) of purified rmIL-1Ra, commercial mIl-1Ra, or commercial hIL-1Ra. Thereafter, the mIL-2 concentration in the culture supernatant was determined by ELISA. Data are representative of two independent experiments and presented as the mean ± SD. Means denoted by different letters differed significantly (*p* < 0.05). n.d. not detected. Mouse (gmLAB), rmIL-1Ra purified from NZ-IL1Ra; mouse (commercial), commercially available mIl-1Ra; human (commercial), commercially available hIL-1Ra.

The ability of purified rmIL-1Ra to inhibit IL-1 signaling was examined using EL4.NOB-1 cells^22^. IL-2 secretion was observed in EL4.NOB-1 cells stimulated with IL-1β but not in unstimulated cells (Fig. 3). IL-1β-stimulated IL-2 secretion was significantly reduced with increasing concentrations of purified rmIL-1Ra and commercial mIL-1Ra and hIL-1Ra (Fig. 3). The effect of suppressing IL-2 production was stronger in the order of mIL-1Ra, hIL-1Ra, and purified rmIL-1Ra (Fig. 3).

### Intestinal delivery of rmIL-Ra by NZ-IL-1Ra

**Fig. 4:**
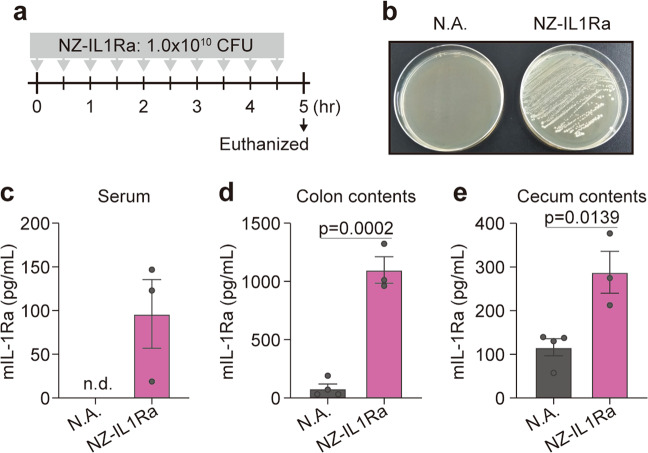
Intestinal delivery of rmIL-Ra by oral administration of NZ-IL1Ra. **a** Schedule for oral administration of NZ-IL1Ra. **b** GM17cm agar plate streaked with colonic contents of the NZ-IL1Ra group or nonadministration group. **c**–**e** mIL-1Ra concentration in the serum, colonic contents, and cecal contents of each group. Data are the mean ± SE (*n* = 3–4), and each dot in the plot represents one mouse. N.A. nonadministration group, NZ-IL1Ra NZ-IL1Ra administration group, n.d. not detected.

NZ-IL1Ra was orally administered every 30 min for 10 doses. Thirty minutes after the last administration, serum and the contents of the cecum and colon were collected (Fig. 4a). In a plating assay, the growth of colonies was confirmed in the NZ-IL1Ra group, and the 16S rRNA region of randomly selected colonies exhibited homology with *L. lactis* (100%) (Fig. 4b). In contrast, no colonies were observed in the nonadministration group (Fig. 4b). The serum mIL-1Ra level was dramatically increased with serial NZ-IL-1Ra administration (Fig. 4c). Furthermore, the mIL-1Ra level in the cecal and colonic contents increased with NZ-IL1Ra administration compared with that in the nonadministration group (Fig. 4d, e).

### Oral administration of NZ-IL1Ra to mice with acute colitis

**Fig. 5:**
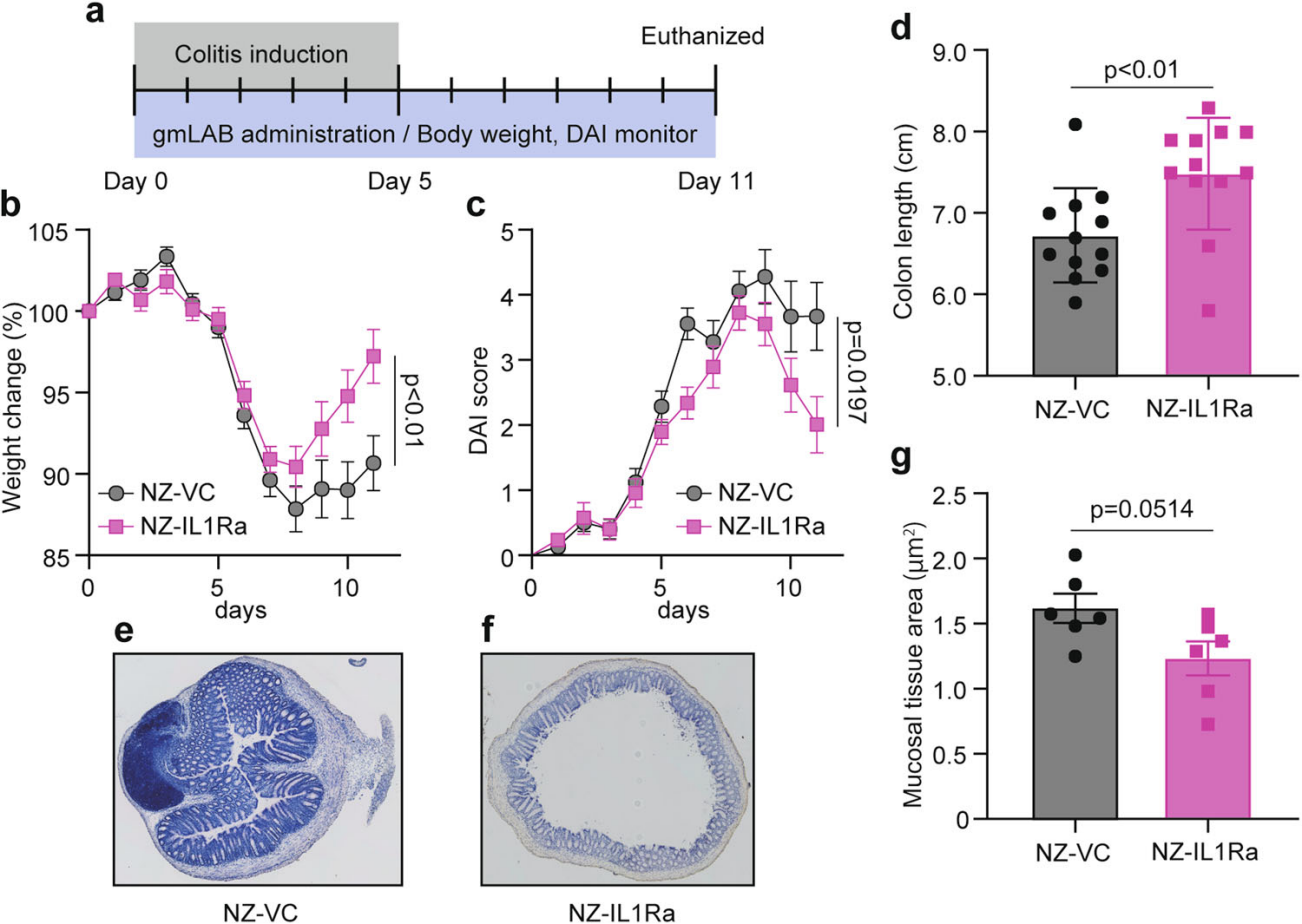
Effect of oral administration of NZ-IL1Ra on DSS-induced colitis in model mice. **a** Schedule for colitis induction and oral administration. The in vivo experiment was repeated 3 times (total *n* = 18). **b**, **c** Change in body weight and DAI score, respectively, of mice with DSS-induced colitis. Data are the mean ± SE (*n* = 18). The *p* values of day 11 results are shown. **d** Colon length on day 11. Data are the mean ± SE (*n* = 12), and each dot in the plot represents one mouse. **e**, **f** Representative images of colon tissue stained with HE (10× magnification). **e** NZ–VC, **f** NZ-IL1Ra. **g** Total mucosal area on day 11. Data are the mean ± SE (*n* = 6), and each dot in the plot represents one mouse.

Mice with DSS-induced colitis were orally administered NZ-IL1Ra or NZ-VC, and the experiment was performed according to the schedule shown in Fig. 5a. Colitis severity was determined by measuring body weight and determining the DAI score daily^16^. On the last day (day 11), the mice were euthanized, the colon was collected, and its length was measured.

In mice allowed to freely drink water containing 3% DSS, body weight decreased significantly from days 5 to 8, and the DAI score increased, confirming that DSS-induced acute colitis (Fig. 5b). After day 8, no increase in body weight was observed in the NZ–VC oral administration group. In contrast, a significant recovery of body weight was observed in the NZ-IL1Ra oral administration group (Fig. 5b). The DAI score also decreased significantly in mice orally administered NZ-IL1Ra (Fig. 5c). NZ-IL1Ra oral administration also significantly improved the colitis-associated shortening of the colon, as measured on the last day of the experiment (Fig. 5d). HE staining of colon sections removed on the last day revealed a decrease in immune cell infiltration in the NZ-IL1Ra administration group and a significant decrease in the total mucosal tissue area (Fig. 5e–g).

### NZ-IL1Ra suppresses CD4^+^ IL-17A^+^ cells and inflammatory cytokines in MLNs and the colon

**Fig. 6:**
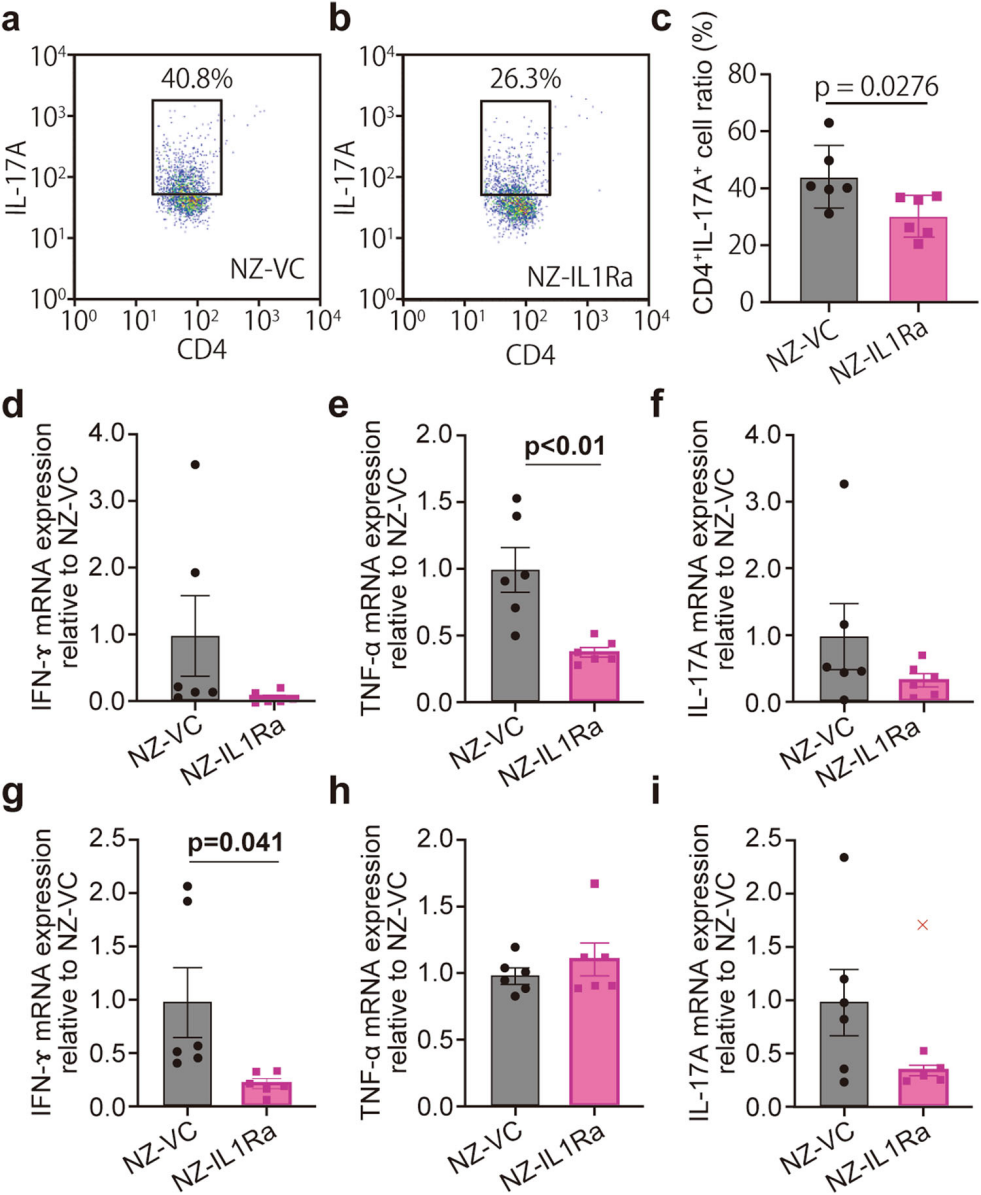
Anti-inflammatory effect of NZ-IL1Ra on cytokine expression. **a**–**c** MLNs excised on day 11 were stained with an Alexa Fluor488 anti-mouse CD4 antibody and a PerCP/Cy5.5 anti-mouse IL-17A antibody. Representative flow cytometry dot plots of CD4^+^ IL-17A^+^ cells in NZ-VC (**a**) and NZ-IL1Ra groups (**b**) are shown. **c** Graph showing the average percentage of CD4^+^ IL-17A^+^ cells. Data are the mean ± SE (*n* = 6), and each dot in the plot represents one mouse. **d**–**i** Graph showing relative expression levels of IFN-γ (**d**, **g**), TNF-α (**e**, **h**), and IL-17A (**f**, **i**) mRNA in the colon and MLNs, respectively. Values are shown relative to the NZ–VC group, and significant differences are indicated by *p* values. Data are the mean ± SE (*n* = 6), and each dot in the plot represents one mouse (“x” notations indicate outliers).

In the IBD model, TNF-α and IFN-γ are overexpressed, and the production of IL-17A by Th17 cells is greatly affected by IL-1 signaling^23–26^. The colon and MLNs were collected and analyzed to investigate the anti-inflammatory effects. The proportion of CD4^+^ IL-17A^+^ cells in MLNs was determined using flow cytometry. The NZ-IL1Ra group exhibited a significant decrease in the proportion of CD4^+^ IL-17A^+^ cells vs. the NZ–VC group (Fig. 6a–c).

Total RNA was extracted from the colon and MLNs, and the expression of TNF-α, IFN-γ, and IL-17A was measured by qPCR (Fig. 6d–i). Excluding TNF-α expression in MLNs, there was a trend toward decreased inflammatory cytokines. Significantly decreased expression of TNF-α in the colon and IFN-γ in the MLNs was observed in the NZ-IL1Ra group (Fig. 6e, g).

### Effects of NZ-IL1Ra on the cecal microbiota in DSS-induced colitis

**Fig. 7:**
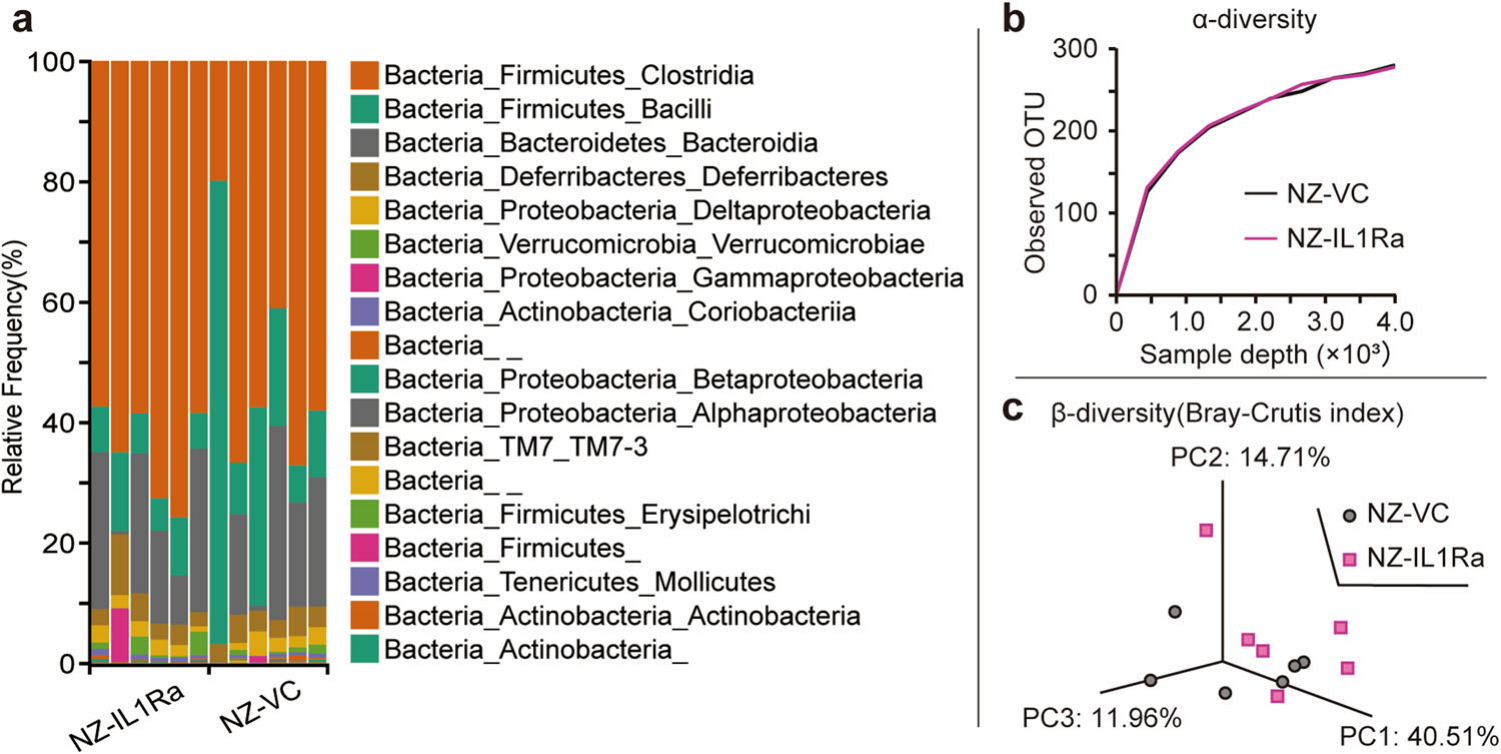
Effects of NZ-IL1Ra on the cecal microbiota. **a** The cecal microbiota composition in the NZ-IL1Ra and NZ–VC groups (*n* = 6). The V3–V4 region of 16S rRNA from cecal contents was amplified and sequenced. The resulting sequences were analyzed using the QIIME2 pipeline and classified using the Greengenes database. **b** α-Diversity (the rarefaction curve of observed OTUs) in the NZ-IL1Ra and NZ–VC groups (*n* = 6). **c** β-Diversity (the Bray–Curtis index) in each group (*n* = 6).

Considering that IBD occurs in the gastrointestinal tract, studies examining effects on the microbiota are critical. Therefore, the cecal microbiota in mice with DSS-induced acute colitis was analyzed using a next-generation sequencing approach. The cecal microbiota compositions in the NZ-IL1Ra and NZ–VC groups are shown in Fig. 7a. There was no significant change in α-diversity (observed OTUs) following the oral administration of NZ-IL1Ra compared with that in the NZ–VC group (Fig. 7b). Similarly, there was no significant change in β-diversity (Bray–Curtis) following the oral administration of NZ-IL1Ra (Fig. 7c).

## Discussion

gmLAB not only produce proteins at low cost but also deliver the proteins produced directly to the intestinal mucosa^10^. Focusing on suppression of IBD by controlling the IL-1 signaling involved in the exacerbation of inflammation, we constructed a gmLAB that secretes rmIL-1Ra and investigated its efficacy using an acute colitis model. As the expression cassette is controlled by the *P*_nisA_ promoter, the addition of nisin, a type of bacteriocin, initiates the transcription of genes downstream of the promoter^27^. Western blotting indicated that nisin-stimulated NZ-IL1Ra to markedly secrete rmIL-1Ra. Interestingly, we found that the amount of secreted IL-1Ra was extremely high (2 mg/L) compared to that of other interleukins for which secretion was found to be controlled by the NICE system in previous studies (Table 1). The expression efficiency of NZ-IL1Ra was also remarkable as an endotoxin-free bacterial factory for IL-1Ra. Although it is unclear why the expression level was so high, these results emphasize the importance of ensuring compatibility between the expression system and target protein in applications of microbial therapeutics.

**Table 1:** Quantity of recombinant interleukin, as determined using the NICE system.

Interleukin	Concentration (µg/L)	Reference
IL-1Ra (mouse)	2000	This study
IL-2 (mouse)	2	^34^
(porcine)	580	^35^
IL-10 (mouse)	40	^36^
IL-12 (mouse)	0.185	^37^
(mouse)	0.065	^38^
IL-18 (mouse)	0.7	^39^
IL-22 (human)	10	^40^
IL-35 (mouse)	–	^41^

We also investigated whether rmIL-1Ra produced by NZ-IL1Ra exhibits biological activity in inhibiting IL-1 signaling. EL4.NOB-1 cells highly express IL-1R and are known to produce IL-2 upon stimulation with IL-1β^22^. Production of IL-2 by EL4.NOB-1 cells stimulated with IL-1β was suppressed by increasing the concentration of rmIL-1Ra purified from NZ-IL1Ra supernatant, commercially available mIL-1Ra, or hIL-1Ra. These results indicate that rmIL-1Ra produced by gmLAB can play an effective role as an endogenous antagonist of IL-1. Inhibition of IL-1 signaling increased in the order of mIL-1Ra, hIL-1Ra, and purified rmIL-1Ra, suggesting that the affinity of gmLAB-secreted rmIL-1Ra for IL-1R declined due to a change in the three-dimensional structure due to cleavage of the signal peptide and/or introduction of the His-tag.

Next, we investigated the delivery of rmIL-1Ra to the intestinal tract by oral administration of NZ-IL1Ra. The results suggested that NZ-IL1Ra reached the colon alive and secreted rmIL-1Ra in situ because *L. lactis* colonies were confirmed in a plating assay, and the mIL-1Ra concentration increased in the contents of the cecum and colon. Furthermore, oral administration of NZ-IL1Ra resulted in a dramatic increase in the serum mIL-1Ra level, which was below the detection limit in the nonadministration group. These results suggested that highly secreted rmIL-1Ra translocated into the blood and that NZ-IL1Ra oral administration could be a useful approach for suppressing IL-1 signaling.

Delivery of IL-1Ra directly to the intestinal tract not only reduces the prevalence and severity of side effects seen with systemic administration but also produces a beneficial anti-inflammatory effect^6^. Indeed, in clinical trials involving the subcutaneous administration of Anakinra (a recombinant hIL-1Ra), serious side effects were reported in 5.1% of patients. By contrast, gmLAB expressing recombinant target protein functions as an inexpensive tool for protein delivery directly to the intestinal tract without the requirement for purification or sophisticated techniques for culture^10^. Therefore, we investigated the anti-inflammatory effect of NZ-L1Ra using an acute colitis model. Oral administration of NZ-IL1Ra from day 0 to day 11 promoted weight gain from day 8 and decreased the DAI score. Furthermore, the results of HE staining of tissue sections indicated a reduction in the thickening of the mucosal tissue area of the colon caused by inflammation, suggesting that orally administered NZ-IL1Ra alleviates acute colitis. In DSS-induced colitis, inflammatory cytokines, such as TNF-α and IFN-γ, are overexpressed and known to be involved in the worsening of colitis symptoms^23^. In the NZ-IL1Ra group, inflammatory cytokine mRNA expression on day 11 was decreased, suggesting that NZ-IL1Ra suppressed the excessive inflammatory response. Notably, Th17 cell proliferation increases in IBD, as characterized by CD4 and IL-17A production, and this expansion of Th17 cells is enhanced by IL-1 signaling^7,25,26^. In this study, we measured the proportion of CD4^+^ IL-17A^+^ cells in MLNs. Compared with those in NZ-VC, CD4^+^ IL-17A^+^ cells decreased in the NZ-IL1Ra administration group. This result suggests that administration of NZ-IL1Ra suppresses IL-1 signaling in vivo and suppresses the increased expansion of Th17 cells. In addition to testing the intestinal delivery of rmIL-1Ra by NZ-IL1Ra oral administration performed in this study, Shigemori et al.^16^ demonstrated that gmLAB transport target proteins to the mucosal tissue of the colon and that oral administration of NZ–VC does not improve DSS-induced colitis. These results suggest that rmIL-1Ra delivery to the colon by oral administration of NZ-IL1Ra alleviates symptoms by inhibiting IL-1 signaling. However, given that *L. lactis* does not colonize the intestinal tract and that 100-fold more IL-1Ra than IL-1 is required to inhibit signaling^28,29^, preadministration or posttreatment with NZ-IL1Ra may not be effective.

Breakdown of the inflammatory immune response balance is known to be a cause of IBD, but changes in the gut microbiota are also an important factor^30^. We therefore collected cecal contents on the last day of in vivo studies and analyzed the cecal microbiota based on 16S rRNA gene sequencing. For diversity analysis, we determined the indices of α-diversity (observed OTUs) and β-diversity (the Bray–Curtis index). However, there was no significant difference with respect to α- or β-diversity. These results suggested that NZ-IL1Ra improves the symptoms of acute colitis by primarily affecting host IL-1 signaling rather than through effects on the microbiota. Our metagenome analysis, however, did not exclude a possible alteration of minor but important microbial species. The anti-inflammatory effect of NZ-IL1Ra via the microbiota should be further investigated.

The increasing number of patients with IBD in rapidly Westernizing developing countries may cause an unprecedented pandemic^1^. Combatting this problem using existing IBD drugs would not only be very expensive but also require additional expensive equipment and technology for production and administration. The use of NZ-IL1Ra, by contrast, does not require protein purification or difficult culture techniques, and the ability to administer NZ-IL1Ra orally makes it an attractive next-generation agent for treating IBD. However, the use of genetically modified organisms involves ethical issues, and adequate monitoring is necessary to control for release into the environment and potential side effects in the body. Although clinical trials examining gmLAB have demonstrated the effectiveness of biological containment strategies and safety in patients, further research is needed to evaluate the effectiveness of gmLAB in treating diseases such as IBD^31–33^.

In conclusion, we successfully constructed gmLAB that hypersecrete bioactive mIL-1Ra. Oral administration of NZ-IL1Ra to acute colitis mice alleviated colitis symptoms and suppressed excessive immune reactions in the intestinal tract and MLNs. These results suggest that rmIL-1Ra reaches the colon via NZ-IL1Ra and inhibits IL-1 signaling. NZ-IL1Ra could be used as an inexpensive and effective tool for the treatment of colitis by targeting IL-1 signaling in the colon. We hope that this study will advance the application of microbial therapeutics.
